# Understanding the Impact of AI Doctors’ Information Quality on Patients’ Intentions to Adopt AI for Independent Diagnosis: Scenario-Based Experimental Study

**DOI:** 10.2196/62885

**Published:** 2025-08-19

**Authors:** Yongmei Liu, Zichun Wang, Bo Peng

**Affiliations:** 1 Business School, Central South University, Xiaoxiang Middle Road, Jiangwan Building, Xiaoxiang Campus of Central South University, Changsha, 410083, China, 86 18419221269; 2 Urban Smart Governance Laboratory, Central South University, Changsha, China

**Keywords:** artificial intelligence, AI doctor diagnosis system, adoption intention, diagnostic transparency, diagnostic argument quality, perceived expertise, information quality, AI doctor, AI technology, disease diagnosis, decision-making, scenario-based, digital consultation, digital health, health informatics

## Abstract

**Background:**

The development of artificial intelligence (AI) systems capable of independent diagnosis offers a promising solution for optimizing medical resource allocation, especially as their diagnostic accuracy can exceed that of some primary medical staff. However, despite these advancements, many patients exhibit hesitancy toward accepting AI technology, particularly for autonomous diagnostic roles. The mechanisms through which the information quality presented by AI doctors influences patients’ intention to adopt them for independent diagnosis remain unclear.

**Objective:**

This study aimed to examine how the information quality of AI doctors influences patients’ intentions to adopt them for independent diagnosis. Specifically, drawing on the elaboration likelihood model, this study seeks to understand how diagnostic transparency (DT) and diagnostic argument quality (DAQ; as aspects of AI-delivered information) affect patients’ intention to adopt artificial intelligence doctors for independent diagnosis (IAID), with these effects being mediated by perceived expertise (PE) and cognitive trust (CT).

**Methods:**

A scenario-based experiment was conducted to investigate the impact of information quality on patients’ adoption intentions. To test the hypotheses, a 2 (DT: low or high)×2 (DAQ: low or high) between-groups experimental design was used. Each experimental group consisted of 60 valid participants, yielding a total of 240 valid responses. Data were analyzed using 2-way ANOVA and partial least squares.

**Results:**

Both DT (β=.157; *P=*.008) and DAQ (β=.444; *P*<.001) significantly positively affected patients’ PE. As the central route, the influence of the experimental manipulation of DAQ (mean_1_ 4.55, SD 1.40; mean_2_ 5.68, SD 0.81; *F*_1,236_=59.701; *P*<.001; ηp^2^=0.202) on PE is more significant than that of DT (mean_1_ 4.92, SD 1.24; mean_2_ 5.31, SD 1.28; *F*_1,236_=7.303; *P*=.007; ηp^2^=0.030). At the same time, PE has a positive impact on CT (β=.845; *P*<.001), and CT also positively affected patients’ IAID (β=.679; *P*<.001). The serial mediation pathway via PE and CT fully mediated the effects of both DT (β=.090; 95% CI 0.017‐0.166) and DAQ (β=.254; 95% CI 0.193‐0.316) on patients’ IAID.

**Conclusions:**

DAQ (central cue) and DT (peripheral cue) influenced patients’ IAID. These effects were fully mediated through a sequential pathway: both cues enhanced PE—with DAQ exerting a significantly stronger effect than DT—which in turn fostered CT, subsequently shaping IAID. Practically, these results highlight that to foster patient adoption, efforts should prioritize enhancing the quality and clarity of AI’s diagnostic arguments, as this pathway more strongly builds PE and, subsequently, CT. This insight is crucial for designing AI doctors that patients will find acceptable and trustworthy for various diagnostic responsibilities.

## Introduction

### Background

Generative artificial intelligence (AI) is rapidly transforming health care, as AI systems evolve from merely supporting human decisions to performing independent diagnoses. For instance, advanced language models like ChatGPT and Med-PaLM (a large language model developed to provide high-quality answers to medical questions) can now interpret medical texts, analyze symptoms, and offer remarkably accurate diagnostic suggestions [1]. A notable example is Tsinghua University’s “Agent Hospital,” an AI-driven digital platform that autonomously manages complete clinical workflows—from patient intake and diagnosis to treatment planning and follow-up [2]. Similarly, Doctronic’s specialized large language model, trained on medical guidelines, demonstrates around 70% diagnostic agreement with human doctors, highlighting AI’s capacity for autonomous operation [3].

While these technological leaps are significantly reshaping health care delivery, a critical challenge persists. Despite AI’s proven abilities and growing sophistication [4], many patients remain hesitant to accept it for independent diagnostic roles [5]. A key reason for this caution is “algorithm aversion”—the common tendency to distrust or prefer human judgment over algorithmic decisions, even when algorithms are more accurate [6,7]. This aversion poses a significant hurdle to the adoption of AI doctors for independent disease diagnosis. Therefore, understanding how to build patient confidence in AI for these autonomous tasks is essential if we are to use AI effectively to improve health care access.

This study suggests that algorithm aversion can be lessened by improving how AI communicates information. People expect doctors to be experts [8-10], and they likely apply similar standards to AI doctors. Research on algorithm aversion indicates that a lack of clarity in AI’s decision-making (poor transparency) or unconvincing explanations (weak argument quality) can worsen negative perceptions [6,7,11,12]. Consequently, our research investigates how enhancing the AI’s diagnostic transparency (DT) and diagnostic argument quality (DAQ) influences patients’ view of its expertise. We believe that improving these aspects of information delivery can lead to a more positive assessment of the AI’s abilities.

Establishing cognitive trust (CT) is also vital in the context of medical AI and can help overcome algorithm aversion. Thus, instead of measuring algorithm aversion directly, this study examines how specific, controllable features of AI-generated information—namely, DT and DAQ—influence patients’ intention to adopt artificial intelligence doctors for independent diagnosis (IAID). We propose that these features affect perceived expertise (PE), which in turn fosters CT. By exploring these relationships, our research aims to uncover how to cultivate more positive views of AI doctors operating independently. This understanding could help reduce algorithm aversion and pave the way for broader acceptance of autonomous AI in health care.

### Prior Research

#### Review on AI Adoption Studies

The burgeoning field of AI has seen its applications permeate a diverse array of domains, prompting a surge in scholarly attention to understand the factors influencing its adoption. In the realm of employee recruitment, Pan et al [13] have identified technological complexity and uncertainty as key determinants shaping companies’ intentions to embrace AI. The hospitality sector, including hotels and dining experiences, has not been immune to this trend. Pelau et al [14] discovered that while AI’s anthropomorphic features alone do not determine user acceptance, the perceived empathy and quality of interaction significantly mediate this relationship, with human-like empathy from AI enhancing user acceptance. Nam et al [15] further elucidated that relative advantage, complexity, and IT expertise are influential in the adoption of AI and robotics within the hotel industry.

The banking industry has also been a focal point of research, with Rahman et al [16] revealing that attitudes toward AI, perceived usefulness, perceived risk, trust, and subjective norms are significant predictors of the intention to adopt AI in banking services.

Medical contexts have garnered particular interest, with studies like Fan et al [17] highlighting trust, social influence, and perceived substitution crises as pivotal in health care professionals’ intentions to adopt AI-based medical diagnosis support systems. Huo et al [18] adopted a unique perspective by examining medical AI acceptance after the service failure, concluding that self-attribution of responsibility by patients enhances trust in AI, thereby improving acceptance for both autonomous and assistive diagnostic treatments, with personality traits playing a moderating role.

Despite these valuable insights into AI adoption in health care, there remains a dearth of exploration into the emergent phenomenon of AI doctors conducting independent consultations. While Uymaz et al [19] have investigated factors such as performance expectancy, perceived task technology fit, high-technology habits, and hedonic motivation affecting physicians’ intentions to use AI doctors, the patient perspective on adopting AI doctors for such independent diagnostic and consultation roles remains significantly underexplored. This literature review underscores the multifaceted nature of AI adoption across various industries and the need for a more granular understanding of user behavior, particularly as AI’s frontiers expand into autonomous decision-making within health care.

#### The Influencing Factors of Algorithm Aversion

Several studies have investigated the effect of algorithm design on algorithm aversion. One salient design factor is the “black box” nature of design, that is, the lack of transparency. When people do not have a clear understanding of how the algorithm works and how it produces decisions, they will exhibit aversion [6,7]. You et al [20] propose a theoretical model based on the judge-advisor system and empirically examine how algorithmic advice affects human judgment compared to the same advice from humans. This effect depends on the level of transparency, which depends on whether and how the prediction performance of the advice source is presented. By making the algorithm transparent, the black box can be unlocked. In turn, transparency can be achieved by making algorithms accessible, interpretable, understandable, interactive, and comprehensive [11].

In addition, argument characteristics, refer to the length of the interpretation and the quality of the interpreted information, also have an impact on algorithm aversion. Gönül et al [12] conducted an experiment of time series plots with short (1‐1.5 lines) and long (4‐5 lines) explanations with varied degrees of information value and found that long explanations and explanations with higher information value were more persuasive.

#### Elaboration Likelihood Model

The elaboration likelihood model (ELM) of persuasion postulates that “important variations in the nature of persuasion are a function of the likelihood that receivers will engage in elaboration of (that is, thinking about) information relevant to the persuasive issue” [21]. ELM theorizes that there are 2 ways of persuasion. The first is the central route, which is controlled by a wide range of information processing aimed at scrutinizing and revealing the core arguments of the issue [22]. In the central route, persuasion is the result of careful and thoughtful consideration of the core argument of the issue, and this route emphasizes how the argument in the persuasive message is understood and processed by the recipient of the argument. The second route is known as the peripheral route and is dominated by nonissue-relevant concerns, also known as the persuasion cues. These persuasive cues are not inherent to the message itself but are relevant to the situation. Under the peripheral route, people focus more on the peripheral factors than on the message itself, and active thinking about informational arguments plays a secondary role in persuasive outcomes.

The main difference between the central route and the peripheral route is the degree of active thinking or cognitive elaboration of the argument. ELM acknowledges that “persuasion can occur at any moment along the elaboration continuum” [21], and active cognitive elaboration produces enduring persuasion outcomes [23]. DT and DAQ play an important role in influencing information reception. Since the DAQ requires the patient to think actively about the diagnosis (ie, the core argument) of the AI doctor, it is taken as the central route. DT, on the other hand, visually reflects information such as accuracy in the form of numerical values, so make it the peripheral route.

### Research Model and Hypotheses

#### The Influence of DT and DAQ on PE

Transparency depends on the degree of disclosure and presentation of algorithm-related information, while DT means that information such as the accuracy of the AI doctor diagnostic system is disclosed at different levels of detail [24,25]. Research on AI collaboration suggests that transparency about why algorithms are trustworthy, and how they work, correlates with an individual’s trust in algorithmic decision aids [26,27]. The transparency of predictive performance enriches the understanding of why algorithms are trustworthy. When AI doctors have higher DT, patients can better understand the principle of AI doctors for disease diagnosis and have a deeper understanding of the prediction accuracy and other information, thus generating a higher PE of AI doctors. Hence, we hypothesize:

- Hypothesis 1: The higher the DT of the AI doctors, the higher the PE of the patients.

An argument is a type of information presentation that seeks to establish the validity of a conclusion by providing reasons or support for it. DAQ refers to the presence and relationships of supporting arguments for the AI doctor’s diagnosis [28,29]. In the past, digital information seeking behavior was modeled as a process involving information quality judgment [30]. Importantly, in this process, argument structure is considered to be a central factor affecting the assessment of information quality [30]. The consequences of trusting and applying poor information in an AI doctor diagnostic system can be severe, so patients will turn to AI doctors for convincing, high-quality health information. When all the necessary components of an argument are present to provide supporting elements for the conclusion, users are more likely to judge that the information provided is relevant, accurate, and useful [30,31]. In the context of this study, when the disease diagnosis conclusion is supported by symptoms and underlying reasons, patients exhibit higher PE toward AI doctors. Hence, we hypothesize:

- Hypothesis 2: The higher the DAQ of the AI doctors, the higher the PE of the patients.

#### The Influence of PE on CT

CT emerges from rational evaluations of a trustee’s competence, reliability, and dependability [32], making it particularly relevant in the context of AI doctor adoption. For patients’ PE of doctors, it is patients’ perception that the doctors are valid medical professionals [33]. When patients recognize AI systems as possessing legitimate medical expertise, they develop rational confidence in the system’s diagnostic reliability—a CT foundation necessary for engagement with AI recommendations. Unlike human doctor interactions where interpersonal elements can foster affective trust, patient interactions with AI systems are primarily transactional, emphasizing the importance of perceived professional capabilities rather than emotional bonds. This cognitive assessment becomes especially critical in health care contexts where patients face uncertainty and vulnerability, yet lack traditional interpersonal cues available in human consultations to form trust judgments. Hence, we hypothesize:

- Hypothesis 3: Patients’ PE of AI doctors has a positive impact on CT.

#### The Influence of CT on IAID

In the context of AI-powered diagnosis, patients with higher CT in AI doctors are more likely to perceive these systems as capable of delivering accurate and unbiased diagnostic outcomes. This perceived competence, in turn, reduces uncertainty and increases patients’ intention to rely on AI in making clinical decisions. Hence, we hypothesize:

- Hypothesis 4: Patients’ CT in AI doctors has a positive impact on IAID.

**Figure 1.**
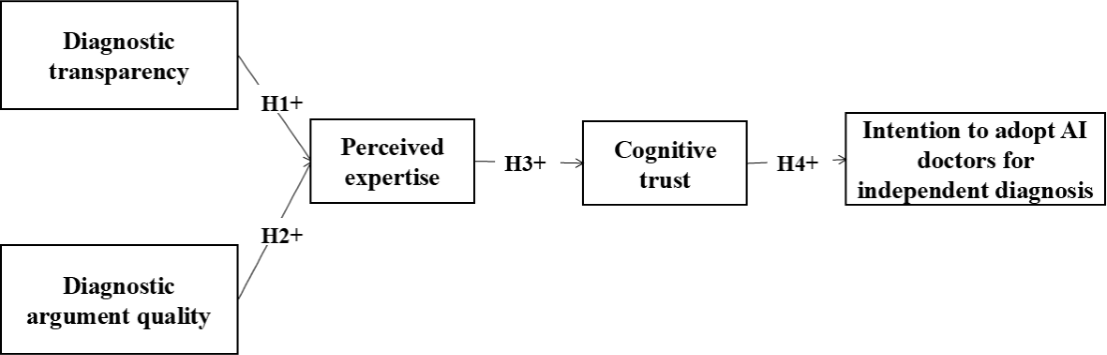
Research model. AI: artificial intelligence; H: hypothesis.

A summary of the conceptual research model is depicted in Figure 1.

## Methods

### Overview

This study used a combined approach to analyze experimental effects, psychometric properties, and hypothesized paths in the model. Specifically, 2-way ANOVA was used to examine the experimental effects, and partial least squares (PLS) was used to assess the psychometric properties of the measures and the causal paths in the proposed model. ANOVA is a traditional method that is often used to test between-group differences and experimental effects, but it is not designed for path analysis or any analysis of psychometric properties of measures.

Partial least squares structural equation modeling (PLS-SEM) is a component-based structural equation modeling approach [34,35] that has gained widespread acceptance for theoretical model testing, particularly in emerging research contexts. We chose PLS-SEM for several reasons. First, it is especially suitable for prediction-oriented research and complex models involving multiple mediating relationships [34,35]. Second, PLS-SEM allows for simultaneous evaluation of measurement and structural models while being robust to nonnormal data distributions, which is common in experimental behavioral research [36,37]. Third, given our research context examining AI doctors where theory is still evolving, PLS-SEM’s capability to handle both theory testing and exploratory research makes it particularly appropriate [34,38]. While PLS is useful for measurement property analysis and model testing, it is not as flexible as ANOVA in conducting multiple comparison tests or testing the relative efficacy of experimental conditions when multiple experimental conditions are involved. Thus, the 2 approaches can be considered complementary, when used together, can provide deeper insights into the phenomenon under study.

### Participant Recruitment

Participants were recruited through the Credamo digital research platform, which allows for targeted sampling based on detailed demographic and experiential criteria. Our inclusion criteria required adult participants with relevant health care experience, ensuring alignment with the study’s focus on AI-mediated medical consultations.

Credamo was selected for its methodological rigor and quality control mechanisms, including identity verification, attention checks, and participant history tracking. These features helped ensure data validity and reliability by minimizing inattentive or ineligible responses. The platform also provided transparent documentation of recruitment procedures and response metrics.

### Target Scenario and AI Application Context

Rather than evaluating a specific commercial AI doctor application, this study used a custom-designed digital consultation scenario to simulate a realistic AI-powered medical interaction. This approach enabled us to control for extraneous variables and systematically manipulate key features of the AI consultation process (eg, response style and diagnostic suggestions), ensuring internal validity and consistency across participants. The simulated scenario was developed based on an extensive review of existing AI medical consultation platforms and expert input from health care professionals, thereby maintaining ecological validity while allowing experimental control.

### Measurement Development

In this study, DT and DAQ were selected as information quality variables for AI doctors. Regarding the DT of AI doctors, in the case of low transparency, AI doctors were introduced using only general language, and no information on diagnostic accuracy was included (eg, AI doctor is an intelligent system that uses AI technology to communicate with patients, which can help patients quickly understand their condition and bring more efficient, accurate, and convenient medical services.). In the case of high transparency, we used precise, numerically detailed language to present information about how AI doctors work and their diagnostic accuracy (eg, AI doctors are based on hundreds of millions of real, complete, and high-quality doctor-patient consultation data to conduct precise and targeted training for large models in the medical and health vertical field. AI doctors are able to diagnose diseases with more than 90% accuracy, human doctors are less than 70% accurate at first diagnosis).

Regarding the DAQ of AI doctors, when the DAQ is low, the “conclusion only” approach is used to obtain the diagnosis of the patient’s disease; when the DAQ is high, the combination of “conclusion plus symptom and reason” is used to get the diagnosis of the patient’s disease. By varying DT at 2 levels (low and high) and DAQ at 2 levels (low and high), our experimental design yielded the following fully crossed four experimental conditions: (1) low DT with low DAQ, (2) low DT with high DAQ, (3) high DT with low DAQ, and (4) high DT with high DAQ.

The study used a scenario-based experimental design structured as a 2×2 between-subjects factorial design, manipulating levels of DT (low or high) and DAQ (low or high). In this study, potential AI doctor users were recruited on the platform, and participants were randomly assigned to 4 different versions of AI doctor introductions and simulated consultation scenarios. The consultation interface consists of 3 rounds of question and answer sessions, in which the patient describes the symptoms they are experiencing. Depending on the experimental conditions, the composition of the answer will change while maintaining the same diagnosis. Specifically, the answer section contains instructions for the patient to describe the symptoms in detail and the disease diagnosis conclusion. In the scenario of low DAQ, the AI doctor concluded: “Based on the symptoms you have provided, we have conducted a preliminary condition analysis and considered a number of possibilities and concluded that you may be facing gastric dyspepsia.” In the scenario of high DAQ, the AI doctor replied, “According to your description, you may be suffering from gastric dyspepsia. Symptoms of stomach pain and nausea half an hour after a meal may be caused by excessive secretion of stomach acid or poor gastrointestinal motility.” The design of all the consultation interfaces is based on real AI doctor consultation situations.

In the process of the experiment, the introduction of the AI doctor was first shown, and participants were required to score according to the DT scale [39], which includes 3 items: “I clearly know how the AI doctor conducts online consultation,” “I have a clear understanding of the principle of the AI doctor’s disease diagnosis,” and “I am completely familiar with the principle of the AI doctor’s disease diagnosis.” Responses were measured on a 7-point Likert scale ranging from 1=strongly disagree to 7=strongly agree. Then, the AI doctor simulated consultation interface was displayed, attention check items were set, and participants were asked to score according to the DAQ scale [36], which includes the following items: “I think the diagnosis of disease provided by the AI doctor is informative,” “helpful,” “valuable,” and “persuasive.” Responses were measured on a 7-point Likert scale ranging from 1=strongly disagree to 7=strongly agree. There is a minimum time limit (2 minutes) for viewing the introduction to the AI doctor and the simulated consultation interface to prevent participants from skipping any pages without paying enough attention. Finally, we measured PE, CT, IAID, AI use experience, and demographic variables.

To ensure good reliability and validity of the questionnaire scale, the measurement items were adapted from the previous literature. All variables were measured by a 7-point Likert scale (1=strongly disagree and 7=strongly agree). PE was adapted from Wu et al [40], and CT was adapted from Komiak and Benbasat [37]. IAID was adapted from Huo et al [18]. Since the interviewees were Chinese, we needed to translate all the items from English to Chinese. All measurements were back-translated by another translator who did not know the background of the study to ensure the accuracy of the translation. The 2 English versions were compared, and potential semantic differences were examined to ensure that the Chinese scales accurately reflected the meaning of all measurements. In total, 10 postgraduates with experience using AI doctors were then invited to participate in a pretest of the scales. Based on their feedback, any ambiguous expressions were modified. The measured constructs and their sources are shown in Multimedia Appendix 1.

The sample size for our 2×2 experimental design was determined through power analysis. We conducted an a priori power analysis using G*Power (version 3.1.9.7; Heinrich Heine University Düsseldorf ) to determine the required sample size. For a 2-way ANOVA with α=.05, power (1−β)=0.80, and anticipating a medium effect size (*f*=0.25) based on prior studies examining technology adoption in health care contexts, the analysis suggested a minimum total sample size of 128. Our sample of 240 total exceeds this requirement, ensuring adequate statistical power. Similar studies such as Kopka et al [41] and Su et al [42] support this approach. To balance the systematic differences in experimental conditions, participants were randomly assigned to each experimental condition.

To ensure the accuracy of the situational experiment design and the participants carefully reading the specific situation, a manipulation check was carried out in this experiment. We used SPSS (version 26.0; IBM Corp) to conduct 2-tailed *t* tests for DT and DAQ scale, finding that the manipulation of DT was effective in producing differences between the 2 conditions (4.73 for low DT and 5.38 for high DT; *t*_238_=−4.874; *P*<.001). Similarly, the manipulation of DAQ was effective in producing perceived differences in the credibility of information across the 2 conditions (4.23 for low DAQ and 5.62 for high DAQ; *t*_238_=−9.614; *P*<.001).

### Statistical Analysis

In this study, we used the confirmatory factor analysis process to test the measurement model. As shown in Table 1, Cronbach α of all constructs is between 0.861 and 0.934. The composite reliability of each construct is between 0.915 and 0.958. These are above the recommended value of 0.7, which means that the measurement model has good reliability [38]. To assess convergent validity, we measured the standard loading of each item as well as the average variance extracted (AVE) for each construct. The results showed that the items’ loadings range from 0.815 to 0.943, these are all much larger than the cutoff value of 0.6 [43]. In addition, the AVE of each construct surpasses 0.5. These results imply that the measurement model has good convergence validity [38].

**Table 1.** Results of confirmatory factor analysis.

Construct and item	Loading	Cronbach α	Composite reliability	AVE^a^
Perceived expertise (PE)		0.905	0.940	0.840
PE1	0.931
PE2	0.902
PE3	0.917
Cognitive trust (CT)		0.861	0.915	0.782
CT1	0.815
CT2	0.903
CT3	0.931
Intention to adopt artificial intelligence doctors for independent diagnosis (IAID)		0.934	0.958	0.883
IAID1	0.943			
IAID2	0.934			
IAID3	0.942			

a AVE: average variance extracted.

Furthermore, as shown in Table 2, the square roots of the AVE of each construct is larger than its correlation coefficients with other constructs, which means that the discriminant validity of the measurement model is confirmed [38]. The experimental variable of DT was coded as 0 for low and 1 for high, and the experimental variable of DAQ was coded as 0 for low and 1 for high.

**Table 2.** Square roots of average variance extracted, and correlations among measured constructs.

Variable	DT^a^	DAQ^b^	PE^c^	CT^d^	IAID^e^
DT					
*r*	1.000	0.000	0.157	0.137	0.075
*P* value	—^f^	>.99	.01	.03	.28
DAQ					
*r*	0.000	1.000	0.444	0.368	0.256
*P* value	>.99	—	<.001	<.001	<.001
PE					
*r*	0.157	0.444	0.917	0.745	0.669
*P* value	.01	<.001	—	<.001	<.001
CT					
*r*	0.137	0.368	0.745	0.884	0.679
*P* value	.03	<.001	<.001	—	<.001
IAID					
*r*	0.075	0.256	0.669	0.679	0.940
*P* value	0.28	<.001	<.001	<.001	—

a DT: diagnostic transparency.

b DAQ: diagnostic argument quality.

c PE: perceived expertise.

d CT: cognitive trust.

e IAID: intention to adopt artificial intelligence doctors for independent diagnosis.

f The diagonal values are the square roots of the average variance extracted. DT and DAQ are binary manipulation variables and not applicable to the Fornell–Larcker criterion.

### Ethical Considerations

Ethics approval for this study was granted by the ethical review board of the Business School at Central South University (approval: CSUBS20240112). All procedures strictly adhered to the relevant provisions of the Personal Information Protection Law of the People’s Republic of China. Prior to participation, all respondents were presented with a digital information sheet outlining the study’s purpose, procedures, confidentiality measures, and their rights. Informed consent was obtained electronically; it was explicitly stated in the information sheet that by completing and submitting the questionnaire, participants were deemed to have read, understood, and voluntarily agreed to participate in the study. Those who did not wish to participate could choose to exit the survey. Participation was entirely voluntary, and participants could withdraw at any time. The questionnaire did not collect any personally identifiable information such as names or contact details. All data were collected and stored in an anonymous format and used solely for the purposes of this study.

## Results

### Overview

A scenario-based experiment was conducted to examine the impact of information quality on patients’ adoption intentions. The study used a between-subjects design with 4 experimental groups, each comprising 60 valid participants. Data collection was carried out over the course of 1 week, yielding a total of 264 completed questionnaires. After removing responses that failed attention checks or were incomplete, 240 valid questionnaires remained, resulting in an effective response rate of 90.9%. Each experimental group retained 60 valid participants. Detailed demographic characteristics of the sample are presented in Table 3.

**Table 3.** Demographic information of respondents (N=240).

Characteristics	Participants, n (%)
Sex	
Male	115 (47.9)
Female	125 (52.1)
Age (years)	
<20	5 (2.1)
20‐29	147 (61.2)
30‐39	60 (25)
40‐49	18 (7.5)
≥50	10 (4.2)
Income (Chinese Yuan**^a^** per month)	
<3000	62 (25.8)
3000‐5999	50 (20.8)
6000‐8999	52 (21.7)
9000‐11,999	44 (18.3)
12,000 and above	32 (13.4)
Education	
High school or below	8 (3.3)
College degree or associate degree	187 (77.9)
Master’s degree or above	45 (18.8)
Occupation	
Student	64 (26.7)
Government and public institutions	9 (3.7)
Business employees	135 (56.3)
Self-employed persons	5 (2.1)
Other	27 (11.2)
AI^b^ use experience (times per week)	
Under 30 minutes	56 (23.3)
30‐60 minutes	53 (22.1)
1‐2 hours	61 (25.4)
2‐4 hours	38 (15.8)
4‐8 hours	22 (9.2)
8 hours and above	10 (4.2)

a A currency exchange rate of ¥1=US $0.14 is applicable.

b AI: artificial intelligence.

### Experimental Group Means

Table 4 reports the mean scores and SDs for PE and CT, grouped by experimental conditions. Among the 4 conditions, the high DT with high DAQ condition showed the highest level of PE and CT. The high DT or high DAQ group reported the highest scores on both PE (mean 5.87, SD 0.57) and CT (mean 5.74, SD 0.76), while the low DT or low DAQ group had the lowest scores (PE: mean 4.34, SD 1.23 and CT: mean 4.58, SD 1.07).

**Table 4.** Descriptive statistics of measured constructs by experimental condition (n=60).

Experimental condition	Perceived expertise, mean (SD)	Cognitive trust, mean (SD)
Low diagnostic transparency with low diagnostic argument quality	4.34 (1.23)	4.58 (1.07)
Low diagnostic transparency with high diagnostic argument quality	5.49 (0.97)	5.27 (1.07)
High diagnostic transparency with low diagnostic argument quality	4.76 (1.53)	4.72 (1.30)
High diagnostic transparency with high diagnostic argument quality	5.87 (0.57)	5.74 (0.76)

To assess the effectiveness of the experimental group assignment, demographic analyses were conducted. One-way ANOVAs revealed no significant differences across the 4 experimental groups for sex (*F*_3,236_=0.280; *P*=.84), AI use experience (*F*_3,236_=0.717; *P*=.54), education level (*F*_3,236_=1.065; *P*=.36), age (*F*_3,236_=1.327; *P*=.27), or income (*F*_3,236_=1.427; *P*=.24). However, a significant difference was observed for occupation (*F*_3,236_=6.078; *P*<.001). Therefore, in the Principal Findings section, we included occupation as a covariate to further examine our conclusions.

### Experimental Effects

We use 2-way ANOVA in SPSS (version 26.0) to test the effects of DT and DAQ on PE. The results showed that DT had a significant positive impact on PE (*F*_1,236_=7.303; *P*=.007; ηp^2^=0.030). Compared with low transparency, high transparency can make patients have higher PE (mean_1_ 4.92, SD 1.24 and mean_2_ 5.31, SD 1.28), hypothesis 1 was supported. DAQ also had a significant positive impact on PE (*F*_1,236_=59.701; *P*<.001; ηp^2^=0.202). Compared with low argument quality, high argument quality can make patients have higher PE (mean_1_ 4.55, SD 1.40 and mean_2_ 5.68, SD 0.81), hypothesis 2 was supported. The interaction term (DT×DAQ) was not significant in any of these ANOVA tests (*F*_1,236_=0.013; *P*=.91; ηp^2^<0.001). After including occupation as a covariate, analysis of covariance results confirmed that the central-route effect of DAQ remained highly significant (*F*_1,235_=58.227; *P*<.001), whereas occupation itself did not yield a significant effect (*F*_1,235_=0.681; *P*=.41). Similarly, the effect of DT on PE remained significant (*F*_1,235_=5.522; *P*=.02), with occupation again showing no significant influence (*F*_1,235_=0.296; *P*=.59).

**Figure 2.**
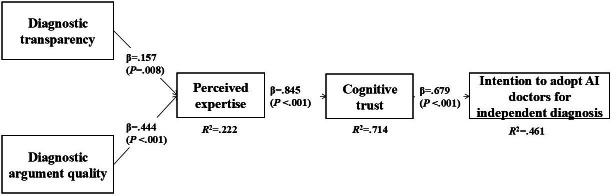
Partial least squares analysis of main effects. AI: artificial intelligence.

SmartPLS (version 4.0; SmartPLS GmbH) was used to test whether the correlation path coefficients were significant, and the results are shown in Figure 2. Hypothesis 1 further confirmed that DT has a significant impact on PE (β=.157; *P*=.008). Hypothesis 2 further confirmed that DAQ has a significant impact on PE (β=.444; *P*<.001). All significant effects previously tested using 2-way ANOVA remain unchanged. In addition, PE significantly positively affected patients’ CT in AI doctors (β=.845; *P*<.001), hypothesis 3 was supported. CT significantly positively affected patients’ IAID (β=.679; *P*<.001), hypothesis 4 was supported. The variances explained by PE, CT, and IAID were 22.2%, 71.4%, and 46.1%, respectively.

To test the mediating effects, we applied the bootstrap method using SmartPLS (version 4.0). A total of 5000 bootstrap resamples were generated to estimate 95% CIs for the indirect and direct effects. According to the results in Table 5, the direct effect of DT on CT or IAID was not significant. Meanwhile, the indirect effects (ie, DT→PE→CT and DT→PE→CT→IAID) were significant. It means that PE plays a fully mediating role between DT and CT, and PE→CT plays a fully mediating role between DT and IAID. The same applies when DT is replaced by DAQ. The direct effect of PE on IAID was significant, and the indirect effect (ie, PE→CT→IAID) was also significant. This means that the effect of PE on IAID was partially mediated by CT.

**Table 5.** The results of the mediation effect test.

Indirect path	Coefficient	95% CI	Direct path	Coefficient	95% CI	Result
DT^a^→PE^b^→CT^c^	0.133	0.025 to 0.241	DT→CT	0.005	−0.064 to 0.073	Full
DT→PE→CT→IAID^d^	0.090	0.017 to 0.166	DT→IAID	−0.018	−0.113 to 0.079	Full
DAQ^e^→PE→CT	0.377	0.294 to 0.455	DAQ→CT	−0.010	−0.085 to 0.072	Full
DAQ→PE→CT→IAID	0.254	0.193 to 0.316	DAQ→IAID	0.007	−0.095 to 0.107	Full
PE→CT→IAID	0.338	0.182 to 0.509	PE→IAID	0.332	0.116 to 0.523	Partial

a DT: diagnostic transparency.

b PE: perceived expertise.

c CT: cognitive trust.

d IAID: intention to adopt artificial intelligence doctors for independent diagnosis.

e DAQ: diagnostic argument quality.

## Discussion

### Principal Findings

The purpose of this study is to explore whether the DT and DAQ of AI doctors influence PE, thereby affecting the CT in AI doctors, and ultimately affecting the IAID. The results of this study found that both variables significantly positively affected patients’ PE of AI doctors. The mean analysis of the experimental group also found that “high DT with high DAQ” showed the highest level of PE and CT.

The analysis of variance showed that, as the central route, the influence of the experimental manipulation of DAQ on PE is more significant than that of DT. In line with these findings, results from SmartPLS further demonstrated that the path coefficient for the effect of DAQ on PE was notably higher than that of DT.

These results collectively suggest that when patients process AI doctors’ diagnoses through the central route—particularly when the DAQ is high—they are more likely to perceive the AI doctor as possessing greater expertise. Thus, the quality of the diagnostic argument plays a more crucial role in shaping PE than transparency alone.

At the same time, PE significantly positively affected patients’ trust in AI doctors and played a full mediating role in the effects of DT and DAQ on CT. CT also positively affected patients’ IAID, and DT and DAQ exert their influence on IAID exclusively through the sequential mediation of PE and CT. CT only partially mediated the effects of PE on IAID.

### Theoretical Implications

We advance prior studies on the adoption of AI in health care by delving into the mechanism through which the information quality of AI doctors shapes patients’ PE, a critical antecedent to CT and the subsequent adoption of AI for independent diagnostic roles [44-46]. While existing literature has established the significant role of PE in fostering CT and the adoption of AI-based medical systems and highlighted the importance of perceived usefulness and ease of use [47,48], the specific influence of AI system design elements—such as DT and argument quality—on the formation of PE for AI performing independent diagnoses remains underexplored from a mechanism design perspective. Our research addresses this gap by examining how these distinct dimensions of information quality impact patients’ perceptions of the AI doctor’s expertise when considered for autonomous diagnostic tasks.

Our central theoretical contribution elucidates the differential effects of DT (a peripheral cue) and DAQ (a central cue) on patients’ PE regarding AI doctors. We demonstrate that argument quality exerts a significantly stronger positive influence on this PE. This finding highlights that the persuasiveness of the AI’s reasoning plays a more critical role than mere transparency in shaping perceptions of expertise, which subsequently fosters CT and strengthens patients’ IAID diagnosis.

By unpacking the full sequential mediation—from information cues to PE, to CT, and ultimately to adoption—our study offers a nuanced understanding of the cognitive mechanisms driving acceptance of autonomous AI. This pathway not only clarifies how patients form CT in AI doctors but also provides a concrete strategy for mitigating algorithm aversion. Specifically, our results underscore the importance of presenting strong, evidence-based diagnostic arguments as a key lever for enhancing PE and CT, thereby promoting patients’ intention to embrace AI in autonomous clinical roles.

### Practical Implications

Our findings offer several important implications for health care practitioners, AI system designers, and health service platform administrators dealing with AI intended for independent diagnostic roles. For digital intelligent consultation platforms using AI for autonomous diagnosis, our results provide evidence-based guidance for optimizing AI doctor interface design. This involves prioritizing the presentation of high-quality diagnostic arguments over mere transparency features. This suggests that platform designers should invest resources in developing clear, evidence-backed explanations for AI-generated independent diagnoses rather than focusing solely on technical transparency mechanisms. Health care administrators can leverage these insights to enhance patient engagement with AI diagnostic tools intended for independent use by ensuring that consultation outputs emphasize clinical reasoning and evidence-based support for these diagnoses.

From a patient education perspective, our research highlights the need for targeted communication strategies that build PE in AI systems designed for independent diagnosis through quality information provision. Health care providers should focus on educating patients about the clinical reasoning capabilities and evidence base of these AI systems, particularly when they are used for making autonomous diagnostic decisions, rather than overwhelming them with technical details about algorithms. This approach can help mitigate algorithm aversion and facilitate the integration of such AI tools into patient health care decision-making processes where AI-driven independent diagnosis is an option.

For policy makers and health care regulators, our findings underscore the importance of establishing standards for information quality in AI systems that perform independent diagnoses rather than solely focusing on technical accuracy metrics. Regulations should ensure that AI consultation platforms offering independent diagnostic services provide high-quality diagnostic arguments that meet patients’ cognitive processing needs. This will facilitate informed decision-making and increase the likelihood of successful adoption of AI for independent diagnostic tasks in health care settings. This practical framework can guide the development of certification standards for AI diagnostic systems intended for autonomous diagnosis, ensuring that they effectively support patient autonomy while improving health care access and efficiency.

### Limitations of the Study

While this study has yielded noteworthy conclusions, it is not without its limitations that warrant future exploration. First, the selection of antecedent variables for algorithm aversion was confined to transparency and argument quality. Subsequent research could adopt a more holistic approach to these influencing factors, thereby enriching the theoretical framework of antecedent variables. Second, the age range of the sample population was predominantly 20‐49 years. This narrow age spectrum may introduce bias into the empirical analysis and does not adequately represent the perspectives of the older people demographic on AI doctors. Third, the data collection was synchronized to a single time point, which could potentially skew the results. In medical research, it is crucial to assess patients’ long-term adoption intentions and behavioral states to gain a comprehensive understanding of their engagement with AI diagnostic systems. Finally, because our sample had a relatively homogeneous educational background, our findings may be influenced by this characteristic and thus primarily applicable to populations with similar educational profiles. Future research with more diverse samples, including individuals with lower levels of education, is needed to determine whether these results extend to the broader population.

### Conclusions

In this study, we investigated the influence of the central and peripheral routes of information quality from AI doctors on users’ intentions to adopt such systems. The mediating roles of PE and CT within these pathways were also examined. Data were analyzed using 2-way ANOVA and PLS, and the empirical findings substantiated all proposed hypotheses. The results underscore the critical importance of high DT and robust DAQ in fostering patient adoption. Within the realm of medical AI, it is imperative to intensify focus on the interplay among information quality, individual perception, and adoption behavior. The outcomes of this research offer strategic insights and recommendations for the refinement and prospective evolution of AI diagnostic systems, particularly from the vantage point of digital intelligent consultation platforms. Additionally, the study highlights the necessity of incorporating a broader spectrum of factors that contribute to algorithm aversion as antecedent variables to unravel diverse mechanisms underpinning adoption decisions.

## Supplementary material

10.2196/62885 Multimedia Appendix 1 Measurement scales.

## Abbreviations

AI: artificial intelligence
AVE: average variance extracted
CT: cognitive trust
DAQ: diagnostic argument quality
DT: diagnostic transparency
ELM: elaboration likelihood model
IAID: intention to adopt artificial intelligence doctors for independent diagnosis
PE: perceived expertise
PLS: partial least squares
PLS-SEM: partial least squares structural equation modeling

## References

[1] Wang D, Zhang S (2024). Large language models in medical and healthcare fields: applications, advances, and challenges. Artif Intell Rev.

[2] (2025). China unveils world’s first fully AI-powered hospital. Medbound.

[3] Zimmerman W This health startup is harnessing AI for quick, free diagnoses—with shocking accuracy. New York Post.

[4] Duan Y, Edwards JS, Dwivedi YK (2019). Artificial intelligence for decision making in the era of Big Data—evolution, challenges and research agenda. Int J Inf Manage.

[5] Longoni C, Bonezzi A, Morewedge CK (2019). Resistance to medical artificial intelligence. J Consum Res.

[6] Dzindolet MT, Pierce LG, Beck HP, Dawe LA (2002). The perceived utility of human and automated aids in a visual detection task. Hum Factors.

[7] Kayande U, De Bruyn A, Lilien GL, Rangaswamy A, van Bruggen GH (2009). How incorporating feedback mechanisms in a DSS affects DSS evaluations. Inf Syst Res.

[8] Grundnig JS, Steiner-Hofbauer V, Drexler V, Holzinger A (2022). You are exactly my type! The traits of a good doctor: a factor analysis study on public’s perspectives. BMC Health Serv Res.

[9] Dopelt K, Bachner YG, Urkin J, Yahav Z, Davidovitch N, Barach P (2021). Perceptions of practicing physicians and members of the public on the attributes of a “Good Doctor”. Healthcare (Basel).

[10] Kotzee B, Ignatowicz A, Thomas H (2017). Virtue in medical practice: an exploratory study. HEC Forum.

[11] Chander A, Srinivasan R, Chelian S, Wang J, Uchino J Working with beliefs: AI transparency in the enterprise.

[12] Gönül MS, Önkal D, Lawrence M (2006). The effects of structural characteristics of explanations on use of a DSS. Decis Support Syst.

[13] Pan Y, Froese F, Liu N, Hu Y, Ye M (2022). The adoption of artificial intelligence in employee recruitment: the influence of contextual factors. Int J Hum Resour Manage.

[14] Pelau C, Dabija DC, Ene I (2021). What makes an AI device human-like? The role of interaction quality, empathy and perceived psychological anthropomorphic characteristics in the acceptance of artificial intelligence in the service industry. Comput Human Behav.

[15] Nam K, Dutt CS, Chathoth P, Daghfous A, Khan MS (2021). The adoption of artificial intelligence and robotics in the hotel industry: prospects and challenges. Electron Markets.

[16] Rahman M, Ming TH, Baigh TA, Sarker M (2023). Adoption of artificial intelligence in banking services: an empirical analysis. Int J Emerg Mark.

[17] Fan W, Liu J, Zhu S, Pardalos PM (2020). Investigating the impacting factors for the healthcare professionals to adopt artificial intelligence-based medical diagnosis support system (AIMDSS). Ann Oper Res.

[18] Huo W, Zheng G, Yan J, Sun L, Han L (2022). Interacting with medical artificial intelligence: integrating self-responsibility attribution, human–computer trust, and personality. Comput Human Behav.

[19] Uymaz AO, Uymaz P, Akgül Y, Uymaz AO, Uymaz P, Akgül Y (2024). The shift from disease-centric to patient-centric healthcare: assessing physicians’ intention to use AI doctors. Environ Soc Psychol.

[20] You S, Yang CL, Li X (2022). Algorithmic versus human advice: does presenting prediction performance matter for algorithm appreciation. J Manag Inf Syst.

[21] O’Keefe Daniel J (2002). Persuasion: Theory and Research.

[22] Petty RE, Briñol P (2011). Handbook of Theories of Social Psychology.

[23] Petty RE (2018). Attitudes and Persuasion: Classic and Contemporary Approaches.

[24] Lage I, Chen E, He J (2019). An evaluation of the human-interpretability of explanation. arXiv.

[25] Poursabzi-Sangdeh F, Goldstein DG, Hofman JM, Wortman Vaughan JW, Wallach H Manipulating and measuring model interpretability.

[26] Cai CJ, Winter S, Steiner D, Wilcox L, Terry M (2019). “Hello AI”: uncovering the onboarding needs of medical practitioners for human-AI collaborative decision-making. Proc ACM Hum-Comput Interact.

[27] Wolf C, Blomberg J (2019). Evaluating the promise of human-algorithm collaborations in everyday work practices. Proc ACM Hum-Comput Interact.

[28] Petty RE, Cacioppo JT, Goldman R (1981). Personal involvement as a determinant of argument-based persuasion. J Pers Soc Psychol.

[29] Boller GW, Sway JL, Munch JM (1990). Conceptualizing argument quality via argument structure. Adv Consum Res.

[30] Rieh SY (2002). Judgment of information quality and cognitive authority in the web. J Am Soc Inf Sci Technol.

[31] Ricco RB (2008). The influence of argument structure on judgements of argument strength, function, and adequacy. Q J Exp Psychol (Hove).

[32] Legood A, van der Werff L, Lee A, den Hartog D, van Knippenberg D (2023). A critical review of the conceptualization, operationalization, and empirical literature on cognition‐based and affect‐based trust. J Manage Stud.

[33] Ohanian R (1990). Construction and validation of a scale to measure celebrity endorsers’ perceived expertise, trustworthiness, and attractiveness. J Advert.

[34] Fornell C, Bookstein FL (1982). Two structural equation models: LISREL and PLS applied to consumer exit-voice theory. J Market Res.

[35] Chin WW (1998). Modern Methods for Business Research.

[36] Bao Z, Wang D (2021). Examining consumer participation on brand microblogs in China: perspectives from elaboration likelihood model, commitment–trust theory and social presence. J Res Interact Mark.

[37] Komiak SYX, Benbasat I (2006). The effects of personalization and familiarity on trust and adoption of recommendation agents. MIS Q.

[38] Fornell C, Larcker DF (1981). Evaluating structural equation models with unobservable variables and measurement error. J Mark Res.

[39] Zhou L, Wang W, Xu DJ, Liu T, Gu J (2018). Perceived information transparency in B2C e-commerce: an empirical investigation. Inform Manag.

[40] Wu T, Deng Z, Chen Z, Zhang D, Wu X, Wang R (2019). Predictors of patients’ loyalty toward doctors on web-based health communities: cross-sectional study. J Med Internet Res.

[41] Kopka M, Schmieding ML, Rieger T, Roesler E, Balzer F, Feufel MA (2022). Determinants of laypersons’ trust in medical decision aids: randomized controlled trial. JMIR Hum Factors.

[42] Su JM, Huang WL, Huang HC, Tseng YL, Li MJ (2024). A scenario-based web app to facilitate patient education in lung tumor patients undergoing video-assisted thoracoscopic surgery: development and usability testing. Digit Health.

[43] Bagozzi RP, Yi Y (1988). On the evaluation of structural equation models. J Acad Mark Sci.

[44] Asan O, Bayrak AE, Choudhury A (2020). Artificial intelligence and human trust in healthcare: focus on clinicians. J Med Internet Res.

[45] Orbán F, Stefkovics Á (2025). Trust in artificial intelligence: a survey experiment to assess trust in algorithmic decision-making. AI Soc.

[46] Chen A, Zhang Y, Feng N (2025). Thinking twice and weighing outcomes: comparing the dual influence of intelligent healthcare features on word-of-mouth. Behav Inf Technol.

[47] Roy M, Jamwal M, Vasudeva S, Singh M (2024). Physicians behavioural intentions towards AI-based diabetes diagnostic interventions in India. J Public Health (Berl).

[48] Jagemann I, Wensing O, Stegemann M, Hirschfeld G (2024). Acceptance of medical artificial intelligence in skin cancer screening: choice-based conjoint survey. JMIR Form Res.

