# Supplementary material for: Understanding the Impact of AI Doctors’ Information Quality on Patients’ Intentions to Adopt AI for Independent Diagnosis: Scenario-Based Experimental Study
Source: J Med Internet Res. 2025 Aug 19;27:e62885. doi: 10.2196/62885 (PMC12364428; doi:10.2196/62885)
Supplement: Multimedia Appendix 1 [file jmir-v27-e62885-s001.doc]

**Appendix A**

Measurement Scales

| **Construct** | **Measures** | **Sources** |
| --- | --- | --- |
| Perceived expertise | The AI doctor is capable of diagnosing diseases. | Wu et al [1] |
|  | The AI doctor has expertise in disease diagnosis. |  |
|  | The AI doctor provides professional disease diagnosis results. |  |
| Cognitive trust | The AI doctor has the professional ethics of a doctor. | Komiak and Benbasat [2] |
|  | The AI doctor is trustworthy. |  |
|  | The AI doctor's diagnosis is reassuring. |  |
| Intention to adopt AI doctors for independent diagnosis | I am willing to accept when my condition is diagnosed entirely by AI and the human doctor is not involved in the process. | Huo et al [3] |
|  | I accept that the diagnosis of disease is made solely by AI doctors and no further communication with human doctors. |  |
|  | I accept fully following the advice given by AI doctors and taking action. |  |

**References**

1. Wu T, Deng Z, Chen Z, Zhang D, Wu X, Wang R. Predictors of Patients’ Loyalty Toward Doctors on Web-Based Health Communities: Cross-Sectional Study. J Med Internet Res 2019 Sep 3;21(9):e14484. doi: 10.2196/14484

2. Komiak SYX, Benbasat I. The Effects of Personalization and Familiarity on Trust and Adoption of Recommendation Agents. MIS Quarterly Management Information Systems Research Center, University of Minnesota; 2006;30(4):941–960. doi: 10.2307/25148760

3. Huo W, Zheng G, Yan J, Sun L, Han L. Interacting with medical artificial intelligence: Integrating self-responsibility attribution, human–computer trust, and personality. Computers in Human Behavior 2022 Jul 1;132:107253. doi: 10.1016/j.chb.2022.107253
